# Prevalence of Non-Alcoholic Fatty Liver Disease and Its Impact on Fibrosis Risk in Inactive Chronic Hepatitis B Patients: Insights from a Cross-Sectional Study

**DOI:** 10.3390/jcm13164738

**Published:** 2024-08-12

**Authors:** Said A. Al-Busafi, Amna S. Al Balushi, Halima H. Al Shuaili, Dalia A. Mahmood, Abdullah M. Al Alawi

**Affiliations:** 1 Department of Medicine, College of Medicine and Health Sciences, Sultan Qaboos University, Muscat 123, Oman; 2 Internal Medicine Program, Oman Medical Specialty Board, Muscat 130, Oman; 3 Department of Medicine, Armed Forces Hospital, Muscat 112, Oman; 4 General Practice, Oman International Hospital, Muscat 333, Oman; 5 Department of Medicine, Sultan Qaboos University Hospital, Muscat 123, Oman

**Keywords:** chronic hepatitis B, non-alcoholic fatty liver disease, prevalence, risk factors, metabolic syndrome, liver fibrosis

## Abstract

**Background:** Chronic hepatitis B (CHB) and non-alcoholic fatty liver disease (NAFLD) are significant causes of chronic liver disease, potentially leading to liver cirrhosis and hepatocellular carcinoma. Moreover, the coexistence of CHB and NAFLD is increasingly common, although the relationship between NAFLD and inactive CHB infection remains poorly understood. **Objectives:** This study aimed to investigate the prevalence of NAFLD among patients with inactive CHB, identify risk factors for NAFLD, and determine predictors of significant fibrosis in these patients. **Methods:** This single-center cross-sectional study targeted patients with inactive CHB at Sultan Qaboos University Hospital from January 2010 to November 2021. **Results:** A total of 425 patients with inactive CHB were identified, of which 53.1% were male and 62.6% were aged 40–60 years. The prevalence of NAFLD was 47.8%. Various independent factors were associated with NAFLD, including type 2 diabetes mellitus, elevated low-density lipoprotein levels, high hemoglobin levels, low platelet counts, and normal alpha-fetoprotein levels. Significant associations were noted between NAFLD and significant fibrosis, with 10.5% of CHB patients with NAFLD exhibiting significant fibrosis compared to 1.4% of those without NAFLD. Other significant parameters included male gender, increased age, high alanine transaminase levels, elevated hemoglobin, and decreased platelet levels. **Conclusions:** The high prevalence of NAFLD in patients with inactive CHB and its associations with increased fibrosis and cirrhosis risk underscore the need for comprehensive management strategies for these patients.

## 1. Introduction

Chronic hepatitis B (CHB) infection is a global health concern, affecting an estimated 257.5 million people worldwide [1]. In Oman, the prevalence of CHB carriers is approximately 2.5% [2]. Inactive CHB infection, also termed hepatitis B e-antigen (HBeAg)-negative CHB infection, is diagnosed based on specific criteria: the presence of HBeAg antibodies (anti-HBeAg), undetectable or low hepatitis B virus (HBV) DNA levels, and normal alanine transaminase (ALT) levels [3]. Non-alcoholic fatty liver disease (NAFLD) is another rising liver concern globally [4]. Among Asian individuals with CHB, the prevalence of NAFLD ranges from 14% to 67%, similar to the rates in Western countries [5,6]. In Oman, the prevalence of NAFLD increased from 8.9% in 1990 to 19.5% in 2017, with an estimated annual rise of 2.12% [7]. Another study in Oman reported an even higher prevalence of 67.3% [8].

Over the past few decades, the obesity epidemic has led to NAFLD becoming one of the most significant chronic liver diseases both regionally and globally, particularly given its association with insulin resistance [9,10,11]. Indeed, experts have proposed replacing the term with metabolic dysfunction-associated fatty liver disease (MASLD) to better reflect the metabolic underpinnings of the disease [12]. Unlike NAFLD, which necessitates the exclusion of other causes of liver disease, a diagnosis of MASLD requires evidence of steatosis and at least one metabolic risk factor like obesity or insulin resistance [12]. This new terminology has already been adopted in the latest guidelines from the American Association of the Study of Liver Disease [13].

Various imaging techniques can be used to diagnose NAFLD, such as ultrasonography, computed tomography, and magnetic resonance imaging, while transient elastography can be utilized to assess hepatic fibrosis. Liver biopsy remains an option when other diagnostic methods are inconclusive [14]. While most individuals with NAFLD have no inflammation, those with both fat and inflammation in the liver―a condition known as non-alcoholic steatohepatitis (NASH)―face an increased risk of fibrosis and cirrhosis [14]. As the prevalence of NAFLD rises, so does its cooccurrence with inactive CHB infection [15,16]. While the detrimental effects of active CHB on the liver are well understood, the potential interaction between NAFLD and inactive CHB warrants further investigation. The relationship between these conditions remains unclear, with some research suggesting their coexistence may worsen liver damage and increase the risk of cirrhosis and fibrosis [17].

The current study, the first of its kind in the region, aimed to determine the prevalence of NAFLD among patients with inactive CHB, identify associated risk factors, and determine predictors of significant fibrosis. Understanding local disease patterns is crucial, particularly considering the rising obesity rates that predispose individuals to NAFLD, which can lead to liver cirrhosis and complications such as hepatocellular carcinoma (HCC) and the need for liver transplantation. Examining the interaction between NAFLD and inactive CHB can influence patient management, treatment strategies, and liver disease prognosis. This knowledge has the potential to improve patient outcomes, tailor treatment approaches, and optimize healthcare resource allocation.

## 2. Materials and Methods

This single-center, cross-sectional study enrolled adult patients diagnosed with inactive CHB between January 2010 and November 2021. All participants received regular follow-up care at the Sultan Qaboos University Hospital (SQUH), a tertiary hospital in Muscat, Oman. The inclusion criteria encompassed all adult individuals diagnosed with inactive CHB according to the guidelines of the European Association for the Study of the Liver (EASL) [3]. These criteria included serum anti-HBeAg positivity, undetectable or low levels of HBV DNA (<2000 IU/mL) by polymerase chain reaction testing, and normal ALT levels based on the standard cut-off values. Additionally, the study included some patients diagnosed with inactive CHB who had HBV DNA levels >2000 IU/mL (usually <20,000 IU/mL) accompanied by persistently normal ALT and low fibrosis, according to the EASL guidelines [3]. Participants with missing essential data, such as laboratory or imaging results, were excluded from the study.

The necessary sample size was estimated based on the anticipated prevalence of NAFLD among patients with inactive CHB, aiming for an absolute precision of 5% and a 95% confidence level, resulting in a required sample size of 334. Ultimately, a total of 425 patients with inactive CHB were included in the study. These patients were categorized into case or control groups based on the presence or absence of NAFLD. Ultrasound findings were used to diagnose NAFLD according to the EASL criteria, with evidence of steatosis by imaging or histology after the exclusion of secondary causes, such as increased alcohol intake, viral hepatitis, or steroid use [18].

The relevant data were extracted from the patients’ electronic medical records using the hospital information system at SQUH (TrakCare, InterSystems Corp., Cambridge, MA, USA). Baseline data included demographic characteristics such as age, gender, weight, height, and body mass index (BMI), as well as any medical history of hypertension (HTN), type 2 diabetes mellitus (T2DM), dyslipidemia, or metabolic syndrome (MetS). The latter condition was defined as the presence of any three of the following criteria: central or visceral obesity (waist circumference of ≥102 cm or ≥40 inches in males and ≥88 cm or ≥35 inches in females), elevated triglycerides (≥150 mg/dL or ≥1.7 mmol/L or specific treatment), reduced high-density lipoprotein cholesterol levels (<1.04 mmol/L or <40 mg/dL in males and <1.29 mmol/L or <50 mg/dL in females or specific treatment), elevated blood pressure (BP)(systolic BP of ≥130 mmHg or diastolic BP of ≥85 mmHg or specific treatment for HTN), and elevated fasting plasma glucose (≥5.6 mmol/L or ≥100 mg/dL or previously diagnosed T2DM) [19,20].

Laboratory data included the hepatitis B surface antigen, HBeAg, anti-hepatitis B e-antigen, hepatitis B surface antibody, hepatitis B core antibody, HBV viral load, alpha-fetoprotein (AFP), gamma-glutamyl transferase, alkaline phosphatase, ALT, aspartate aminotransferase (AST), hemoglobin (Hb), platelet (PLT) count, high-density lipoprotein (HDL), total cholesterol, triglycerides, low-density lipoprotein (LDL), and glycated Hb levels. Systolic and diastolic BP readings were also documented. Liver ultrasonography with simultaneous two-dimensional shear wave elastography was performed using the GE LOGIQ E9 XDclear 2.0 ultrasound machine (GE Healthcare, Milwaukee, WI, USA) every six months as part of the routine HCC screening. The ultrasound findings were used to assess for NAFLD and any signs of cirrhosis. The Fibrosis-4 (FIB-4) index, a validated, non-invasive scoring system based on laboratory tests, was used to estimate the degree of liver fibrosis, aiding in the decision of whether a liver biopsy was necessary [21]. FIB-4 index scores were calculated using the MDCalc (MDCalc Calculator. Retrieved from https://www.mdcalc.com/calc/2200/fibrosis-4-fib-4-index-liver-fibrosis (accessed on 26 June 2023)).

Collected data were analyzed using the Statistical Package for the Social Sciences (SPSS) software, version 26 (IBM Corp., Armonk, NY, USA). Categorical variables were presented as percentages and frequencies, while continuous variables were reported as either means with standard deviations or medians with interquartile ranges, as appropriate. Prevalence was expressed as a proportion with a 95% confidence interval (CI). Crude associations were evaluated using odds ratio (OR), a chi-squared test, and a Mann–Whitney *U* test. Multivariate analysis was conducted using logistic binary regression, including variables from the initial analysis with a *p*-value < 0.20. To address potential multicollinearity issues, the Variance Inflation Factor (VIF) was calculated for each predictor variable. Variables with a VIF greater than 10 were considered to exhibit multicollinearity and were excluded from the final model. The level of statistical significance was set at *p* < 0.05.

This study received ethical approval from the institutional Medical Research and Ethics Committee of the College of Medicine and Health Sciences at Sultan Qaboos University. All study procedures were conducted in accordance with the principles of the revised Helsinki Declaration.

## 3. Results

Of the 425 patients with inactive CHB infection enrolled in the study, the majority were male (53.1%) and 40–60 years old (62.6%). The mean BMI was 29.14 ± 6.32 kg/m^2^, with 75.6% classified as overweight or obese. In terms of comorbidities, MetS was present in 14.6% of participants, while 14.6% and 12.5% had T2DM and HTN, respectively. Dyslipidemia was documented in 16.9% of cases. Detectable HBV DNA levels exceeding 2000 IU/mL were found in 5.6% of patients, while elevated AFP levels were noted in 14.5%. Liver enzymes were mildly elevated, with 6.9% and 3.1% of patients showing increased ALT and AST levels, respectively. Additional baseline characteristics of the study population are summarized in Table 1.

The prevalence of NAFLD was 47.8% (95% CI: 43.1–52.5%). Overall, the prevalence of NAFLD was higher in male patients (55.6%, 95% CI: 49.1–62.1%) compared to female patients (39.2%, 95% CI: 32.4–46.0%). Moreover, a higher prevalence was noted among older patients. Interestingly, the prevalence of NAFLD was similar between patients with and without MetS. Table 2 presents a detailed comparison of NAFLD prevalence according to gender, age, and MetS status.

**Figure 1:**
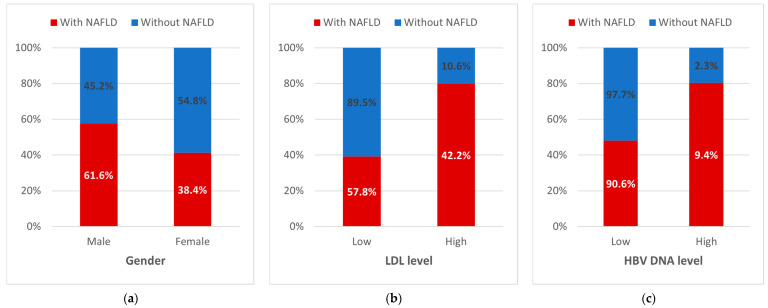
Distribution of NAFLD cases by (**a**) gender, (b) LDL level, and (**c**) HBV DNA level. HBV: hepatitis B virus; LDL: low-density lipoprotein; NAFLD: non-alcoholic fatty liver disease.

Various factors were found to be associated with NAFLD, as shown in Table 3. Gender was significantly correlated (*p* < 0.001), with female patients being 0.52 times less likely to develop NAFLD than males (95% CI: 0.35–0.76). Additionally, significant associations were observed between NAFLD and both LDL levels (OR: 6.187, 95% CI: 3.22–11.90; *p* < 0.001) and HBV DNA levels (OR: 0.223, 95% CI: 0.082–0.609; *p* = 0.002). Figure 1a–c display the frequency of NAFLD according to gender, LDL, and HBV DNA groups.

Furthermore, patients with high ALT levels were significantly more likely to have NAFLD (OR: 10.846, 95% CI: 3.229–36.425) compared to those with normal levels. Conversely, high levels of AFP significantly reduced the likelihood of NAFLD (OR: 0.277, 95% CI: 0.148–0.521; *p* < 0.001). Significant correlations were also found between NAFLD and both the Hb level and PLT count (*p* < 0.001 each). However, in the multivariate regression analysis, only T2DM, high LDL levels, normal AFP levels, elevated Hb levels, and lower PLT counts remained independent predictors of NAFLD. Specifically, the likelihood of NAFLD was 2.606 times higher for patients with T2DM (95% CI: 1.091–6.225) and 4.204 times higher for patients with elevated LDL levels (95% CI: 1.936–9.126). Table 4 details the results of the multivariate analysis.

As per their FIB-4 index scores, most patients (94.3%) were classified as unlikely to have significant fibrosis (score < 1.3; 95% CI: 92.1–9 6.5%). A smaller proportion (4.7%) had scores indicating possible fibrosis (score 1.3–2.67; 95% CI: 2.7–6.7%), while only 0.9% were classified as having advanced fibrosis (score > 2.67; 95% CI: 0–1.8%). The overall prevalence of significant fibrosis, indicated by a FIB-4 index score greater than 1.3, was 5.7% (95% CI: 3.5–7.9%), with cirrhosis observed in 3.3% of cases (95% CI: 1.6–5.0%). The preliminary analysis revealed a significant association between NAFLD and significant fibrosis, with 10.5% of the NAFLD group exhibiting significant fibrosis compared to only 1.4% of patients without NAFLD (OR: 8.564, 95% CI: 2.51–29.18; *p* < 0.001). Moreover, there was a strong association between NAFLD and cirrhosis, with NAFLD patients being 15.15 times more likely to have cirrhosis (95% CI: 1.96–116.66; *p* = 0.001).

Table 5 outlines the associations between various factors and the fibrosis status based on the patients’ FIB-4 index scores. Gender showed a significant correlation with fibrosis, with female patients being less likely to develop significant fibrosis (OR: 0.356, 95% CI: 0.138–0.915) compared to males. High ALT levels also demonstrated a significant association with fibrosis (OR: 5.44; 95% CI: 1.97–15.00). Additionally, increased age, elevated Hb levels, and decreased PLT counts were significantly associated with fibrosis status.

## 4. Discussion

Chronic liver diseases like CHB and NAFLD carry considerable risks, potentially leading to life-threatening consequences such as liver cirrhosis and HCC [22,23]. In particular, NAFLD has emerged as the most prevalent chronic liver disease globally [10]. Despite the increasingly common coexistence of CHB and NAFLD [17], the relationship between NAFLD and inactive CHB infection remains poorly understood. This study aimed to investigate the prevalence of NAFLD in patients with inactive CHB, its associated risk factors, and predictors of fibrosis in this population.

In our study, 53.1% of participants were male. This is consistent with other studies, including those involving long-term vaccinated individuals, which have similarly reported higher rates of CHB infection in males compared to females [24,25]. This gender disparity is largely attributed to the influence of sex hormones. Testosterone can suppress certain immune responses, potentially hindering the effective control of HBV, whereas estrogen is known to have a protective effect against the virus [26]. Furthermore, men may engage more frequently in high-risk behaviors, such as intravenous drug use and sexual relations with multiple partners, thereby increasing their likelihood of HBV exposure [27].

In terms of age distribution, the majority (62.6%) of CHB patients in our study were between 40 and 60 years old. This demographic pattern can be attributed to the widespread availability of the HBV vaccine in Oman. Starting in August 1990, the vaccine was recommended for newborns, followed by school-based campaigns targeting adolescents aged 11–18 years from 2000 to 2005 (i.e., the 1982–1990 birth cohorts) [28]. Despite these efforts, achieving full vaccination coverage across the population took time, potentially leaving some individuals susceptible to infection. Furthermore, infection rates remained elevated among individuals born before the introduction of the vaccine but within the catch-up campaign period. This is partly due to many individuals in this group receiving the vaccine without prior screening for HBV infection [28]. The prolonged duration of the catch-up campaigns could also have contributed to increased infection risk among unvaccinated children exposed to infected family members.

Our study also indicated that 75.6% of patients with inactive CHB infection were overweight or obese, a finding of significant concern amid the global rise in obesity [29]. In 2021, over half of the Omani population was overweight or obese, with 30% having a BMI over 30 kg/m^2^ [30]. Various factors, such as lifestyle changes, sedentary behavior, urbanization, and socioeconomic status, contribute to these high rates [30,31]. This trend is particularly concerning among CHB patients, as obesity may exacerbate metabolic comorbidities and liver disease progression [32,33]. This may stem from several factors, including reduced physical activity and metabolic disturbances associated with liver disease [33]. While a direct link between CHB and obesity remains unclear, the presence of obesity in CHB patients reflects broader societal trends influenced by factors like poor diet and physical inactivity [32]. Although CHB itself does not cause obesity, its interaction with obesity can significantly impact liver disease outcomes [32,33,34,35].

In our study, MetS was present in 14.6% of participants overall, with a similar prevalence (14.8%) among those with NAFLD. Factors such as ethnicity, age, gender, lifestyle, and other population characteristics influence the prevalence of MetS in CHB patients. In Oman, the MetS prevalence ranges widely from 21% in healthy adults to 45.9% in prediabetic populations [36,37,38]. While the MetS rate in our study was lower than that of the general population, international studies suggest that individuals with CHB may face a higher risk of MetS [39,40]. Nonetheless, the precise relationship between MetS and CHB infection remains complex and inconclusive [41]. Some studies suggest a potential link, while others do not find a significant association [42,43]. Evidence indicates that HBV might influence metabolic profiles and MetS development, although the findings vary [39]. In HBV patients, those with MetS often exhibit higher viral loads, with high and low extremes of cholesterol levels correlating with higher HBV DNA levels, indicating a potential interaction with lipid metabolism [44]. Moreover, CHB infection can lead to various liver diseases, indirectly impacting metabolic health [45]. Conversely, a large Taiwanese study reported an inverse association between CHB infection and MetS development, even after adjusting for the BMI, suggesting unique metabolic influences compared to other liver diseases [35]. Thus, while some evidence suggests an interaction between HBV and metabolic factors, the association with MetS remains uncertain.

In addition, 14.6% of our patients were affected by T2DM, which is lower than the 20.4% prevalence observed among HBV-infected patients in Egypt [46]. In Oman, the prevalence of diabetes varies, with age-adjusted rates ranging from 10.4% to 21.1% [47]. The International Diabetes Federation reported an 11.8% prevalence of T2DM among adults in Oman in 2021, below that found in the HBV population [48]. In contrast, a large, community-based study from 2017 showed an overall T2DM prevalence of 15.7%, with prediabetes at 11.8% [49]. Additionally, 35.1% of Omani men have been reported to have impaired fasting glucose, a precursor to T2DM [47]. The relationship between CHB infection and T2DM is complex. A meta-analysis by Cai et al. indicated that HBV-infected patients have a higher risk of developing T2DM compared to non-HBV-infected patients, although the researchers did not differentiate the stages of HBV infection [50]. Chronic inflammation caused by HBV infection may contribute to insulin resistance and impaired glucose metabolism, key factors in T2DM development [51]. This relationship is influenced by age, ethnicity, duration of HBV infection, and other risk factors like obesity or a family history of T2DM [52].

Overall, the prevalence of NAFLD in our study was 47.8%, aligning with rates reported in studies of CHB patients from Iran and Malaysia (46.8% and 47.9%, respectively) [53,54]. The high prevalence of obesity and elevated BMI in the general Omani population likely contribute to this high prevalence of NAFLD among individuals with inactive CHB infection [31]. However, the occurrence of NAFLD in our population was still lower than that of the general Omani population (67.3%) [8]. This finding supports the literature suggesting that CHB patients have a lower prevalence of NAFLD due to the virus impairing fat formation in the liver [55]. We also identified several factors associated with the development of NAFLD in our univariate analysis, including male gender, high LDL levels, high HBV DNA levels, normal AFP levels, high ALT levels, high Hb levels, and low PLT counts. However, in the multivariate analysis, only T2DM, high LDL levels, high Hb levels, decreased PLT counts, and normal AFP levels remained independent factors associated with NAFLD.

Our study showed that male gender was not an independent risk factor for NAFLD. This finding contrasts with other studies that have identified male gender as significantly associated with liver steatosis among both CHB and non-CHB patients [56,57,58,59]. Two systematic reviews and meta-analyses corroborate that the male gender is a strong risk factor for hepatic steatosis in CHB patients, consistent with the general population [56,57]. Moreover, the prevalence of NAFLD tends to be higher in males, regardless of the presence of inactive CHB infection [60]. This may be attributed to the protective effect of estrogen against hepatic steatosis or fat accumulation in the liver [61].

However, we identified significant relationships between NAFLD and both high LDL levels and T2DM, aligning with previous studies [40,45,48,62]. This is due to the underlying pathogenesis, insulin resistance, which contributes to both NAFLD and T2DM [63,64,65]. Additionally, the LDL receptor, implicated in HBV infection, presumably acts as a viral attachment receptor, thereby altering the LDL levels in inactive CHB [66]. Moreover, HBV may modify hepatic cholesterol metabolism by increasing LDL receptor expression and 3-hydroxy-3-methylglutaryl-coenzyme A reductase via the pre-S1 domain of the viral envelope [67,68]. Given that low HDL cholesterol levels predict poor outcomes in patients with HBV-related liver failure and decompensated cirrhosis [69,70], these findings emphasize the relevance of monitoring lipid values in individuals with inactive CHB.

We also observed independent associations between NAFLD and both low PLT counts and high Hb levels. The etiology of a low PLT count in NAFLD remains unclear, with the proposed factors including hypersplenism, bone marrow hypoplasia, decreased peripheral blood cell survival, and thrombopoietin insufficiency [71,72,73,74]. High Hb levels in CHB patients, as observed in other studies [75,76,77,78], may be due to chronic inflammation, insulin resistance, and hypoxia-induced erythropoietin production. Coexisting conditions such as obesity and MetS may also contribute to elevated Hb levels [79]. Our research also showed a crude association between high ALT levels and NAFLD, although this did not hold in the multivariate analysis. Indeed, ALT, a surrogate marker for liver inflammation, is typically normal in mild cases of NAFLD and inactive CHB [80,81]. Elevated ALT levels indicate the need for further assessment of the hepatitis activity or the progression of NAFLD to more severe forms, such as NASH or liver fibrosis [82]. These results suggest that ALT may act as a cofactor, rather than an independent factor, for NAFLD.

Intriguingly, we observed that patients with inactive CHB and normal AFP levels exhibited a higher prevalence of NAFLD compared to those with high AFP levels. This finding offers a novel perspective on the relationship between AFP levels and NAFLD in patients with inactive CHB, contrary to the prevailing understanding linking elevated AFP levels with hepatic steatosis development [83,84,85,86,87]. For instance, Xu et al. suggested that AFP may act as a cofactor in NAFLD development, albeit not an independent factor [83]. Other researchers have reported a correlation between serum AFP levels and NAFLD grade, with higher AFP levels potentially indicating more severe NAFLD, even though liver enzymes ALT and AST do not show such correlations [86]. Additionally, elevated AFP levels have been associated with the histopathological findings in NAFLD patients, suggesting a potential link between AFP levels and liver pathology [85]. However, our study presents an interesting deviation from these established patterns.

This discrepancy suggests that the mechanisms underlying NAFLD in the context of inactive CHB might differ from those in other hepatic conditions. Several hypotheses could explain our findings. In inactive CHB, normal AFP levels may indicate a less aggressive disease state, allowing for the development of NAFLD without the concurrent increase in AFP typically seen in more active or advanced liver disease. Alternatively, the metabolic profile of patients with inactive CHB might differ in ways that influence both AFP production and lipid metabolism, leading to this unique association. Our findings underscore the need for further research to elucidate the pathophysiological mechanisms linking the AFP levels and NAFLD in inactive CHB. Understanding these interactions could have significant implications for patient management, potentially guiding more tailored and effective treatment strategies. Given the mixed findings in the literature regarding AFP and NAFLD, our study contributes to a more nuanced understanding of liver disease progression in specific patient populations, highlighting the importance of context in interpreting biomarker data.

In our study, substantial links were found between NAFLD and fibrosis or cirrhosis, particularly in patients with inactive CHB, with an increased risk in NASH patients [88,89,90]. The joint existence of NAFLD and CHB accelerates disease progression due to metabolic abnormalities, oxidative stress, and chronic inflammation. Variable fibrosis prevalence exists based on disease severity, individual characteristics, and additional risk factors. The major determinants identified include gender, age, ALT and Hb levels, and PLT counts, with women, especially premenopausal, presenting lower fibrosis risks, potentially due to sex hormone influence [91,92,93,94,95,96,97]. A heightened risk exists for postmenopausal women. Age is another significant contributor, with susceptibility increasing due to prolonged virus exposure [98,99]. Elevated ALT and Hb levels were found to connect to escalated liver inflammation and possible red blood cell turnover and systemic inflammation, linking to fibrosis [100,101,102,103,104,105].

High ALT levels were also significantly associated with fibrosis in our study, consistent with the literature findings that link ALT with increased hepatic inflammation and fibrosis [100]. Studies across various populations have shown that patients with elevated ALT levels have significantly higher odds of fibrosis, reinforcing the utilization of ALT measurements as a reliable marker for liver fibrosis in CHB patients [101,102]. Elevated Hb levels have also been associated with higher fibrosis stages, suggesting a potential link between increased red blood cell turnover and liver fibrosis, although studies confirming this association are scarce [103,104]. Potential mechanisms by which elevated Hb levels might contribute to liver fibrosis include increased red blood cell turnover and the resultant heme oxygenase activity, leading to the production of profibrotic cytokines and growth factors. Elevated Hb might also reflect the underlying systemic inflammation and oxidative stress, which contribute to fibrosis progression [103]. However, studies on NAFLD patients have reported conflicting results regarding this association [75,105].

Finally, we found decreased PLT counts to be significantly associated with fibrosis in our population. Thrombocytopenia, defined as a PLT count of less than 150 × 10^3^ per µL, is frequently seen in advanced liver disease and can indicate portal hypertension and splenic sequestration, both common in liver fibrosis. The pathophysiology behind this association involves the role of PLTs in liver regeneration and repair. Yang et al. showed that the PLT count could predict significant liver fibrosis in patients with CHB infection [106]. Huang et al. also confirmed that a decreased peripheral PLT count is associated with liver fibrosis in both chronic hepatitis B and C patients, highlighting the commonality of this marker across different hepatitis infections [107]. A comprehensive review by Parikh et al. emphasized the importance of the PLT count as a non-invasive marker for assessing liver fibrosis in CHB patients, thereby aiding in the early detection and management of fibrosis, potentially improving patient outcomes [108]. Overall, the consistent association between thrombocytopenia and liver fibrosis underscores the importance of monitoring PLT counts in CHB patients to effectively assess and manage the fibrosis risk.

Our study has several limitations that should be considered when interpreting the results. Firstly, the cross-sectional design precludes the establishment of causality between NAFLD and fibrosis in patients with inactive CHB. Longitudinal studies are needed to confirm these findings and understand the temporal relationship between these conditions. Secondly, our study was conducted at a single center, which may limit the generalizability of the results to other populations. The sample population was primarily drawn from patients attending SQUH, and regional variations in disease prevalence and risk factors may not be fully captured. Thirdly, the reliance on non-invasive methods for diagnosing NAFLD and fibrosis, such as ultrasonography and FIB-4 score, may introduce measurement biases compared to histological confirmation via liver biopsy. Lastly, potential confounding factors, such as lifestyle habits and detailed metabolic profiles, were not comprehensively assessed, which could influence the observed associations. Future research should address these limitations by incorporating multicenter cohorts, prospective designs, and comprehensive data collection to enhance our understanding of the interplay between NAFLD and CHB infection.

## 5. Conclusions

Our study highlights the significant impact of NAFLD on the risk of fibrosis among inactive CHB patients. The high prevalence of NAFLD in this population and its association with increased fibrosis and cirrhosis risk underscore the need for comprehensive management strategies that address both conditions concurrently. Moreover, we identified several key factors independently associated with NAFLD, including T2DM, high LDL and Hb levels, low PLT counts, and normal AFP levels. In turn, significant fibrosis was associated with male gender, increasing age, and high ALT levels. These findings emphasize the importance of closely monitoring and managing metabolic factors and liver health in patients with inactive CHB to prevent disease progression. Future studies should examine how the MASLD criteria, including the presence of metabolic risk factors, refine our understanding of the link between inactive CHB and liver disease progression. This revised terminology reflects the growing recognition of the metabolic underpinnings of this condition, potentially offering a more nuanced understanding of how liver disease develops in patients with inactive CHB.

## Figures and Tables

**Table 1:** General characteristics of the study sample.

Characteristic	*n* (%)
Gender (*n* = 424)	
Male	225 (53.1)
Female	199 (46.9)
Age in years (*n* = 425)	
<40	136 (32.0)
40–60	266 (62.6)
>60	23 (5.4)
BMI in kg/m^2^ (*n* = 209)	
<25	51 (24.4)
25–30	79 (37.8)
>30	79 (37.8)
Comorbidities (*n* = 425)	
T2DM	62 (14.6)
HTN	53 (12.5)
MetS	62 (14.6)
Dyslipidemia	72 (16.9)
Laboratory variables	
Total cholesterol in mmol/L (*n* = 286) *	4.63 ± 1.02
HbA1C in % (*n* = 233) *	5.80 ± 1.36
Hb level in g/dL (*n* = 233) ^†^	12.60 (11.40–13.70)
PLT count in x109/L (*n* = 421) ^†^	277.00 (224.00–336.50)
Albumin level in g/L (*n* = 423) *	42.63 ± 23.45
LDL in mmol/L (*n* = 284)	
Normal (≤2.59)	203 (71.5)
High (>2.59)	81 (28.5)
HBV DNA level in IU/mL (*n* = 425)	
Undetectable/Low (≤2000)	401 (94.4)
High (>2000)	24 (5.6)
AFP level in KIU/L (*n* = 417)	
Normal (≤7)	355 (85.1)
High (>7)	62 (14.9)
GGT level in U/L (*n* = 34)	
Normal (6–42)	31 (91.2)
High (>42)	3 (8.8)
ALT level in U/L (*n* = 423)	
Normal (≤33)	394 (93.1)
High (>33)	29 (6.9)
AST level in U/L (*n* = 423)	
Normal (≤32)	410 (96.9)
High (>32)	13 (3.1)

* Reported as the mean ± standard deviation. ^†^ Reported as the median (interquartile range). AFP: alpha-fetoprotein; ALT: alanine aminotransferase; AST: serum aspartate aminotransferase; BMI: body mass index; GGT: gamma-glutamyl transferase; HBV: hepatitis B virus; Hb: hemoglobin; HbA1C: glycated hemoglobin; HTN: hypertension; LDL: low-density lipoprotein; MetS: metabolic syndrome; PLT: platelet; T2DM: type 2 diabetes mellitus.

**Table 2:** Prevalence of NAFLD according to the subgroup.

Subgroup	*n* (%)	95% CI
Gender		
Male	125 (55.6)	49.1–62.1
Female	78 (39.2)	32.4–46.0
Age in years		
<40	55 (40.4)	32.2–48.6
40–60	137 (51.5)	45.5–57.5
>60	11 (47.8)	27.4–68.2
MetS status		
Absent	173 (47.7)	42.6–52.8
Present	30 (48.4)	36.0–60.8

CI: confidence interval; MetS: metabolic syndrome; NAFLD: non-alcoholic fatty liver disease.

**Table 3:** Associations between NAFLD and various factors.

Factor	*n* (%)		*p*-Value
	Without NAFLD (*n* = 222)	With NAFLD (*n* = 203)	
Gender			0.001
Male	100 (45.2)	125 (61.6)	
Female	121 (54.8)	78 (38.4)	
Age in years			0.110
<40	81 (36.5)	55 (27.1)	
40–60	129 (58.1)	137 (67.5)	
>60	12 (5.4)	11 (5.4)	
BMI in kg/m^2^			0.202
<25	28 (28.9)	23 (20.5)	
25–30	31 (32.0)	48 (42.9)	
>30	38 (39.2)	41 (36.6)	
Comorbidities			
T2DM	27 (12.2)	35 (17.2)	0.138
HTN	28 (12.6)	25 (12.3)	0.926
MetS	32 (14.4)	30 (14.8)	0.915
Dyslipidemia	35 (15.8)	37 (18.2)	0.499
Hb level in g/dL *	12.20 (10.90–13.00)	13.350 (12.30–14.50)	<0.001
PLT count in ×10^9^/L *	321.0 (264.0–361.50)	242.0 (205.25–289.75)	<0.001
LDL in mmol/L			<0.001
Normal (≤2.59)	110 (89.4)	93 (57.8)	
High (>2.59)	13 (10.6)	68 (42.2)	
HBV DNA level in IU/mL			0.002
Undetectable/Low (≤2000)	5 (2.3)	19 (9.4)	
High (>2000)	217 (97.7)	184 (90.6)	
AFP level in KIU/L			<0.001
Normal (≤7)	173 (78.3)	182 (92.9)	
High (>7)	48 (21.7)	14 (7.1)	
ALT level in U/L			<0.001
Normal (≤33)	219 (98.6)	175 (87.1)	
High (>33)	3 (1.4)	26 (12.9)	

* Reported as the median (interquartile range). AFP: alpha-fetoprotein; ALT: alanine aminotransferase; BMI: body mass index; HBV: hepatitis B virus; Hb: hemoglobin; LDL: low-density lipoprotein; MetS: metabolic syndrome; NAFLD: non-alcoholic fatty liver disease; PLT: platelet; T2DM: type 2 diabetes mellitus.

**Table 4:** Multivariate analysis showing the relationships between NAFLD and various parameters (*n* = 279).

Factor	OR	95% CI	*p*-Value
Gender			0.448
Male	0.763	0.380–1.533	
Female	Ref	Ref	
Age in years			
<40	0.618	0.145–2.640	0.654
40–60	1.299	0.330–5.115	0.382
>60	Ref	Ref	0.115
T2DM			0.031
Present	2.606	1.091–6.225	
Absent	Ref	Ref	
Hb level	1.395	1.121–1.735	Hb level
PLT count	0.987	0.983–0.992	PLT count
LDL			<0.001
High	4.204	1.936–9.126	
Normal	Ref	Ref	
HBV DNA			0.056
High	5.330	0.959–29.623	
Undetectable/low	Ref	Ref	
AFP level			0.005
Normal	3.991	1.523–10.458	
High	Ref	Ref	
ALT level			0.073
Normal	4.258	0.875–20.721	
High	Ref	Ref	

AFP: alpha-fetoprotein; ALT: alanine aminotransferase; CI: confidence interval; HBV: hepatitis B virus; Hb: hemoglobin; LDL: low-density lipoprotein; OR: odds ratio; PLT: platelet; T2DM: type 2 diabetes mellitus.

**Table 5:** Associations between fibrosis status and various factors.

Subgroup	*n* (%)		*p*-Value
	Without Fibrosis (*n* = 401)	With Fibrosis * (*n* = 24)	
Gender			0.026
Male	205 (51.6)	18 (75.0)	
Female	192 (48.4)	6 (25.0)	
Age in years			<0.001
<40	133 (33.4)	2 (8.3)	
40–60	248 (62.3)	16 (66.7)	
>60	17 (4.3)	6 (25.0)	
BMI in kg/m^2^			0.834
<25	47 (24.6)	4 (25.0)	
25–30	73 (38.2)	5 (31.3)	
>30	71 (37.2)	7 (43.8)	
Comorbidities			
T2DM	56 (14.1)	6 (25.0)	0.142
HTN	47 (11.8)	6 (25.0)	0.058
MetS	56 (14.1)	6 (25.0)	0.172
Dyslipidemia	68 (17.1)	4 (16.7)	0.958
Hb level in g/dL *	12.60 (11.40–13.60)	13.75 (12.60–14.38)	0.014
PLT count in ×10^9^/L*	283.00 (232.50–342.0)	191.00 (157.00–211.75)	<0.001
LDL in mmol/L			0.612
Normal (≤2.59)	189 (71.9)	14 (66.7)	
High (>2.59)	74 (28.1)	7 (33.3)	
HBV DNA level in IU/mL			0.729
Undetectable/low (≤2000)	23 (5.8)	1 (4.2)	
High (>2000)	375 (94.2)	23 (95.8)	
AFP level in KIU/L			0.081
Normal (≤7)	331 (84.4)	23 (95.8)	
High (>7)	61 (15.6)	1 (4.2)	
ALT level in U/L			0.004
Normal (≤33)	375 (94.2)	18 (75.0)	
High (>33)	23 (5.8)	6 (25.0)	

* Defined as a Fibrosis-4 index score of >1.3. AFP: alpha-fetoprotein; ALT: alanine aminotransferase; BMI: body mass index; HBV: hepatitis B virus; Hb: hemoglobin; LDL: low-density lipoprotein; MetS: metabolic syndrome; NAFLD: non-alcoholic fatty liver disease; PLT: platelet; T2DM: type 2 diabetes mellitus.

## Data Availability

Data supporting the reported results are available from the corresponding author upon reasonable request.
